# Energy, Macronutrients and Micronutrients Intake Among Pregnant Women in Lebanon: Findings from the Updated Lebanese National Food Consumption Survey (LEBANON-FCS)

**DOI:** 10.3390/nu16234059

**Published:** 2024-11-26

**Authors:** Rana Mahfouz, Marie-Therese Akiki, Vanessa Ndayra, Rebecca El Khoury, Marise Chawi, Majida Hatem, Lara Hanna-Wakim, Yonna Sacre, Maha Hoteit

**Affiliations:** 1 Department of Nutrition and Food Sciences, Faculty of Arts and Sciences, Holy Spirit University of Kaslik (USEK), Jounieh P.O. Box 446, Lebanon; ranamahfouz@usek.edu.lb (R.M.); marie_therese.h.akiki@net.usek.edu.lb (M.-T.A.); vanessa.m.ndayra@net.usek.edu.lb (V.N.); rebecca.e.elkhoury@net.usek.edu.lb (R.E.K.); marise.r.chawy@net.usek.edu.lb (M.C.); majida.h.hatem@net.usek.edu.lb (M.H.); 2 Department of Agricultural and Food Engineering, School of Engineering, Holy Spirit University of Kaslik (USEK), Jounieh P.O. Box 446, Lebanon; larahanna@usek.edu.lb; 3 Food Sciences Unit, National Council for Scientific Research of Lebanon (CNRS-L), Beirut P.O. Box 11-8281, Lebanon; 4 PHENOL Research Program, Faculty of Public Health, Section 1, Lebanese University, Beirut P.O. Box 6573, Lebanon

**Keywords:** pregnant women, food consumption, food groups, macronutrients, micronutrients, USDA guidelines, Mediterranean diet, Lebanon

## Abstract

Background: Pregnancy is a crucial period for maternal and fetal health, and in Lebanon, where cultural and economic factors influence dietary practices, there is an urgent need to evaluate the food consumption patterns and diet quality of pregnant women. Aim: To evaluate the food consumption patterns, energy intake, as well as macro- and micro-nutrient intake among a nationally representative sample of Lebanese pregnant women aged 18–49 years old. Methods: A cross-sectional study was carried out from March to October 2023, involving 500 pregnant women from all eight Lebanese governorates. Sociodemographic and medical information was gathered, food consumption was evaluated using a validated Food Frequency Questionnaire (FFQ) and three 24-h recall, and anthropometric measurements were recorded. Results: The current population did not meet the USDA healthy pattern recommendations for whole grain, seafood, dairy, nuts, seeds and soy products consumption but exceeded the guidelines for vegetables, meats, poultry, eggs, oils, and refined grains. According to Mediterranean diet guidelines, the sample fell short in recommended intakes for fruits, olives/nuts/seeds, eggs, and olive oil, while surpassing the recommended levels for potatoes, legumes, pulses, sweets, red meat, processed meat, and fish and seafood. None of the participants met the energy requirements for their trimester and age group. In terms of macronutrient intake, the requirements for protein, unsaturated fats, and fiber were not met, while intakes of fats and sugars were exceeded. Regarding micronutrients, the recommended levels were not fully achieved, with particularly low intakes of vitamin D and iodine, as well as inadequate adherence to recommendations for iron, calcium, vitamin A, vitamin E, zinc, and choline. Additionally, a third of the participants did not meet the recommended intakes for folate and vitamin B12. Conclusions: The findings reveal significant dietary inadequacies among the current population, with participants failing to meet essential recommendations for whole grains and key food groups, alongside insufficient energy intake for their trimesters and age groups. Critical micronutrient deficiencies, particularly in vitamin D, iodine, and B vitamins, highlight the urgent need for targeted nutritional interventions and public health initiatives to improve dietary practices among pregnant women in Lebanon.

## 1. Introduction

Maternal diet plays an essential role in fetal growth and in the prevalence of pregnancy complications, such as preeclampsia, hypertension and gestational diabetes [1]. Fat-soluble vitamins, such as Vitamins A and D, play a critical role in fetal growth and development, particularly in vision, immune function, calcium absorption and bone health, contributing to fetal skeletal development. Vitamin E acts as an antioxidant, protecting cells from oxidative stress and supporting immune function, while Vitamin K is important for blood clotting and bone health, helping to prevent hemorrhagic disease in newborns [2]. Water-soluble vitamins are equally crucial. Vitamin B6 aids protein metabolism and cognitive development in the fetus, while folate (B9) is crucial for DNA synthesis and preventing neural tube defects, leading to recommendations for prenatal supplements; Vitamin B12 supports red blood cell formation and neurological development, with deficiency risking anemia and developmental issues, and Vitamin C boosts collagen synthesis and immune function, important for preventing anemia in pregnant women [2].

A systematic review in the Netherlands on 218 reports from 54 studies found evidence that folate and vitamin D dietary intakes were inadequate, and supplementation was needed. Reasons of inadequacies were the low consumption of vegetables, fruits and fish, in addition to the excessive consumption of sugar, salt and alcohol [3]. Moreover, Kiely et al. [4] highlighted the importance of iron, iodine and vitamin D deficiencies. Worldwide prevalence of anemia among pregnant women has been linked to inadequate dietary intake of iron, coupled with lack of abiding by the recommendations. As for iodine, salt fortification has been a suitable strategy to prevent thyroid disorders, in addition to the consumption of milk and dairy products. However, current shifts in dietary patterns are noticing a shift away from these rich iodine sources. Finally, vitamin D deficiencies have been reported worldwide. An inconsistent intake of fatty fish has been observed, which highlights the importance of supplementation and fortification strategies. In addition to folic acid that prevents neural tube defects, and iodine that prevents cretinism, supplementation of calcium has been proposed as a prevention to hypertension among pregnant women with low calcium dietary consumption [5]. A study in Sweden [6] among 567 pregnant women found daily intakes of folate, selenium, iodine and vitamin D that were lower than the recommendations among more than 50% of the sample. Another study in South Africa [7] among 239 pregnant women found that intakes of folate, iron and vitamin D were inadequate for most of the sample. In the United States, among 1392 pregnant women, a study [8] showed that over the past 2 decades, intake of vitamin C, vitamin A and iron decreased, while the intake of vitamin K, magnesium and calcium increased.

In addition to vitamins, macronutrients—carbohydrates, proteins, and fats—are vital for maternal and fetal health. Carbohydrates provide essential energy for the increased metabolic demands of pregnancy, with complex carbohydrates from whole grains, fruits, and vegetables helping to stabilize blood sugar levels. Proteins are vital for tissue growth and repair, crucial for fetal development and maternal tissue formation, necessitating increased intake, especially in the second and third trimesters. Healthy fats, particularly omega-3 fatty acids, support brain development and influence fetal cognitive function [9].

Providing a balanced energy/protein supplemented intake, can increase birthweight among newborns. On the contrary, supplementation with high protein may cause deleterious fetal health outcomes. Regarding carbohydrate intake, attention has been put on the glycemic index or the glycemic load of diet, reflecting the blood glucose level increase after each food consumption. Following a relatively low glycemic index or the glycemic load diet can protect pregnant women from gestational diabetes and prevent infant large-weight infants for their gestational age [5]. In a systematic review and meta-analysis on 54 articles [10], it was reported that total energy intake was lower than the recommended average. Moreover, it was found to be higher in American and Eastern Mediterranean regions. It was also higher in the third trimesters of pregnancies. In the United States, Miketinas et al. [8] showed that over the past two decades, intake of carbohydrates increased, while that of fat and proteins remained stable.

The worldwide westernization of diet shifted nutrition away from healthy choices. In fact, the study in Sweden [6] identified two dietary clusters, one that is relatively healthy, rich in vegetables, fruits, fish and while grains, and the other westernized and unhealthy, being rich in pizza, French fries, meat, soft drinks, snacks and candies. In the African study [7], intakes of fruits and vegetables were inadequate while those of added sugars and sugary drinks were high.

In Lebanon, a country marked by an array of culinary traditions and deep-rooted commitment to the Mediterranean diet, there is a complex interplay of cultural, socio-economic, and environmental determinants that shape pregnant women’s dietary practices [11]. At the same time, rapid globalization, nutrition transition and economic stresses, coupled with high prevalence of food insecurity are rendering pregnant women to be exceptionally nutritionally vulnerable to malnutrition [12]. Systematic reviews highlight that food-insecure women often consume fewer fruits and vegetables, leading to reduced intake of essential vitamins and minerals such as vitamin A, B6, calcium, magnesium, zinc, and folate. Instead, they may rely more on calorie-dense, carbohydrate-rich foods, which can contribute to poor overall nutrition and health outcomes [13].

To the best of our knowledge, there is no comprehensive study that assessed the nutritional intake of Lebanese pregnant women, particularly during the escalating crises. Therefore, this study aims to evaluate food consumption patterns, energy intake, and both macro- and micronutrient intake among a nationally representative sample of Lebanese pregnant women aged 18 to 49 years.

## 2. Methods

### 2.1. Study Design and Eligibility Criteria

This cross-sectional study was conducted over a period of five months, spanning from March to October 2023. It included a nationally representative sample of Lebanese pregnant women. The participants were healthy (didn’t develop gestational diabetes, pre-eclampsia or eclampsia) aged between 18 and 49 years, who were in different trimesters of pregnancy and lived in various districts and altitudes throughout Lebanon.

### 2.2. Sampling Method and Recruitment Process

For the sample to be nationally representative, a minimum sample size of 384 participants was required. The initial population was N = 1,244,000 [14]. It was calculated considering women in reproductive age from 2018 to 2019 in the report done by the Lebanese Republic Central Administration of Statistics, using the following Giezendanner formula: n = (t^2^N)/(t^2^ + (2e)^2^(N − 1)), where “n” represents the sample size, “N” represents the size of the initial population, “t” represents the margin coefficient (1.96) deducted from the level of confidence, “e” represents the margin of error (5%) [15]:

Based on formula developed by the Gizendanner formula (with 95% confidence interval and 5% margin of error), the minimum acceptable sample size was 384 participants.

In this study, the calculation was as follows:

n = 1.96^2^ × 1,244,000/(1.96^2^ + (2*0.05)^2^ (1,244,000 − 1) = 384.12

**Figure 1:**
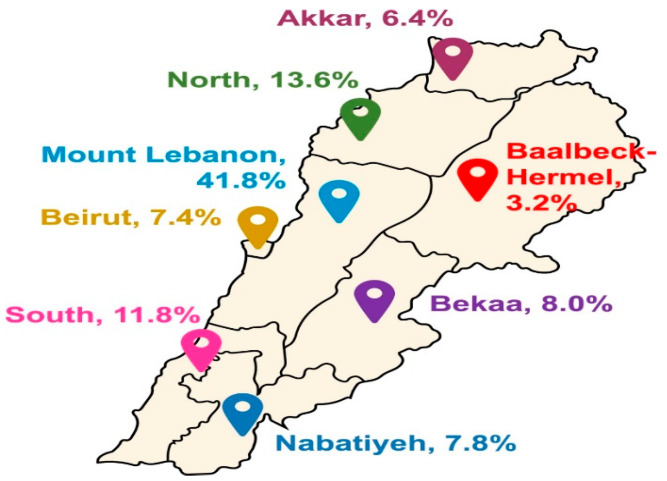
Participant distribution across the Lebanese governorates.

To account for the nonresponse rate and to increase the precision of the estimates, rounding the sample to reach 500 participants was considered, and women from the 8 Lebanese governorates were included. Out of 600 individuals reached, 70 did not respond (11.7% nonresponse rate). The stratified proportional sampling distribution method was used. The process of recruiting participants involved utilizing multiple networks, which included pregnant women identified in private clinics, hospitals, medical centers, and primary health care centers. Gynecologists were cooperative in the initial phases of the study, either by facilitating the process of reaching women and sometimes informing pregnant women about the study and explaining the study objectives or by welcoming the dietitians and agreeing to meet with their patients. Individuals who opted to engage in the study were initially briefed on the nature of the research and subsequently assessed for eligibility. Those who qualified then provided their oral consent, indicating their readiness to participate. Participant distribution across the 8 governorates is shown in Figure 1.

### 2.3. Data Collection

In the initial stage of data collection, interviews were conducted utilizing a pre-tested questionnaire to gather comprehensive information regarding the participants’ health, socioeconomic status, and demographic factors. Participants provided details such as their age, governorate of residence, home size, number of rooms, educational background, employment status, monthly household income, and the presence of diseases. The crowding index (CI), which evaluates a household’s socioeconomic status, was calculated using home size and the number of rooms.

#### 2.3.1. Administration of Food Frequency Questionnaire

Qualified dietitians conducted 30-min interviews with the participants to administer a semi-quantitative validated food frequency questionnaire (FFQ) consisting of 157 items [16]. To aid participants in accurately recalling their dietary intake and estimating portion sizes, they were supplied with clear instructions and visual aids. The interviewers carefully documented the servings, and the frequency of consumption identified as daily, weekly, or monthly, for each food item. The FFQ documented the frequency of food consumption among participants over the last few months, with respondents specifying the number of servings, grams, and the frequency of their intake, whether daily, weekly, or monthly. FFQ categories included “Bread and cereals”, “Potato, pasta, rice and legumes”, “Milk and dairy products”, “Fruits and fruit juices”, “Vegetables”, “Meats, fish and eggs”, “sugars and sweets”, “Salty snacks”, “Dressings, oils and fats” and “beverages”.

#### 2.3.2. Administration of 24-h Recall

The consumption of each individual food and beverage was comprehensively recorded using three 24-h recalls, two on typical weekdays and another on a weekend day. The same strategies were adopted as the FFQ to ensure precise data collection.

#### 2.3.3. Anthropometric and Biometric Measurement

During this stage, participants’ anthropometric measurements, like weight, height, Mid-Upper Arm Circumference (MUAC) and hemoglobin levels were collected. To ensure the reliability of the data, standardized methods and calibrated tools, such as a digital scale for weight and a stadiometer for height. Each participant’s height and weight were measured, where weight was taken in duplicate, and the mean value was considered. The Body Mass Index (BMI) in kg/m^2^ was derived by averaging these measurements. A BMI of 25 or higher was considered as overweight, while a BMI of 30 or above was considered as obese. MUAC, which is an indicator of malnutrition, was measured through a measuring tape. The normal range of MUAC was considered between 22 and 27.6 cm. MUAC levels below 22 or 23 cm indicate severe maternal malnutrition and low birth weight child [17]. As for the Hemoglobin, which is an indicator of anemia, “DiaSpect Tm Hemoglobin Analyzer” (EKF Diagnostics, Barleben, Germany was utilized) [18]. Hemoglobin levels below 11 g/dL indicated anemia [19].

### 2.4. Data Management and Data Analysis

The coding and organization of data were conducted using Excel 2016. Food items were categorized according to two different dietary approaches: the ‘Mediterranean Diet’ and the ‘United States Department of Agriculture Diet’ (‘USDA Diet’). The computation of daily intake for each food group (in g/day) was performed and compared to the relevant dietary guidelines. This involved utilizing data from the Food Frequency Questionnaire (FFQ) to approximate the daily intake per food item. Daily frequency (servings/day) was calculated by dividing the reported weekly or monthly servings by 7 or 30, respectively. Following this, the daily consumption (g/day) was determined by multiplying these daily frequencies by the serving sizes (g/serving). Food items were assigned classifications based on both dietary systems, with the cumulative daily consumption for each food group (in grams) established through the summation of quantities from all items within that category. Upon calculating the daily intake in grams, the energy, macro- and micronutrients, as well as fiber content were assessed using ‘Nutritionist Pro’ software (version 8.1, 2022, First Data Bank, Nutritionist Pro, Axxya Systems, San Bruno, CA, USA). The software provides nutritional analysis for a variety of food items, menu options, and recipes [20]. The nutritional data was compared in accordance with age-specific and trimester-specific Dietary Reference Intakes (DRIs) from the ‘Food and Nutrition Board, Institute of Medicine, National Academies’ (NIH), which encompass the ‘Acceptable Macronutrient Distribution Range’ (AMDR), ‘Adequate Intake’ (AI), and ‘Recommended Dietary Allowance’ (RDAs) [21]. Energy requirements were based on the ‘Dietary Guidelines for Americans 2020–2025’ [22].

### 2.5. Statistical Analysis

The “Statistical Package for the Social Sciences, the IBM SPSS Inc. Statistics” (version 23, New York USA) was used for data analysis, at a 95% confidence interval. The analysis involved determining frequencies (N) and percentages (%) for categorical variables, in addition to calculating means and standard deviations (SD) for continuous variables. Bivariate analysis was conducted to compare nutrient consumption across age groups and trimesters. Comparisons between the two age categories were evaluated using “independent samples *t*-test”, while comparison between the three trimesters categories was done using one-way ANOVA test. A *p*-value ≤ 0.05 was considered significant.

### 2.6. Ethical Considerations

The ethical approval for the current study was granted by the Research Ethical Committee (REC) of the Higher Center for Research (HCR) at the Holy Spirit University of Kaslik (USEK) (13 January 2023). The research was carried out in alignment with the ethical principles established in the Declaration of Helsinki. Prior to participation, individuals provided their informed consent and were made aware that their involvement was voluntary and confidential. Also, they were notified that their involvement was voluntary and can withdraw from the study at any time.

## 3. Results

### 3.1. Socio-Demographic Characteristics of the Population

Results show that most of the study participants (66%) were 30 years old or younger. A significant portion originated from Beirut/Mount Lebanon (49.2%), with others from Northern Lebanon (20%) and Southern Lebanon/Beqaa (30.8%). Following the crisis, over 35.8% of mothers faced a decline in their salaries, yet the vast majority retained a good socioeconomic status (98%) (Table 1).

### 3.2. Food Groups Consumption and Comparison to Different Diets Recommendations

#### 3.2.1. Food Groups Consumption

Women aged 18–30 years had higher consumption of whole grains, refined grains, vegetables, fruits, nuts, seeds and soy products, oils, as well as meat, poultry and eggs. On the contrary, women aged 31–50 years had higher intake of dairy and seafood. Nevertheless, no significant difference was reported between age groups (Table 2).

Among women aged 18–30 years whole grain consumption is significantly higher in the third trimester, compared to the first and second trimesters. Among women aged 31–50 years, fruits consumption was highest in the first trimester, followed by the third then the second trimesters (*p*-value < 0.05) (Table 3). Although difference was not significant, fruits and vegetables intakes were highest in the youngest age group in the first trimester, followed by the second then the third. Among the older group of women, fruit consumption was highest in the third trimester, followed by the first then the second. Regarding vegetables consumption, it was highest in the third trimester, then the second and finally the third. Regarding nuts, seeds and soy products, and dairy intake, among women aged 18–30 years, highest intake was recorded during the second trimester, followed by the third then the first. However, among Women aged 31–50 years, highest nuts, seeds and soy products was in the second, followed by the first and the third. Dairy intake was highest in the second trimester, followed by the first trimester, followed by the third and the second. Oils consumption was highest in the third trimester among the younger women, followed by the third and the second. Among the older women, oils intake was highest in the third trimester, then the first and the second. Regarding meat, poultry and eggs consumption, it was highest in the third trimester among women aged 18–30 years, followed by the second and the first trimesters. On the contrary, among women aged 31–50 years, highest intake was recorded in the first trimester, then the second and the third. As for seafood, the highest intake in the younger group was observed in the second trimester, followed by the first and the third. Among older women, highest intake was in the third trimester, then the second and the first. The difference was not significant. For a comprehensive description of the food items associated with each food group Table S1 can be checked.

#### 3.2.2. Comparison of the Food Groups Consumed to the USDA Diet Recommendations

**Figure 2:**
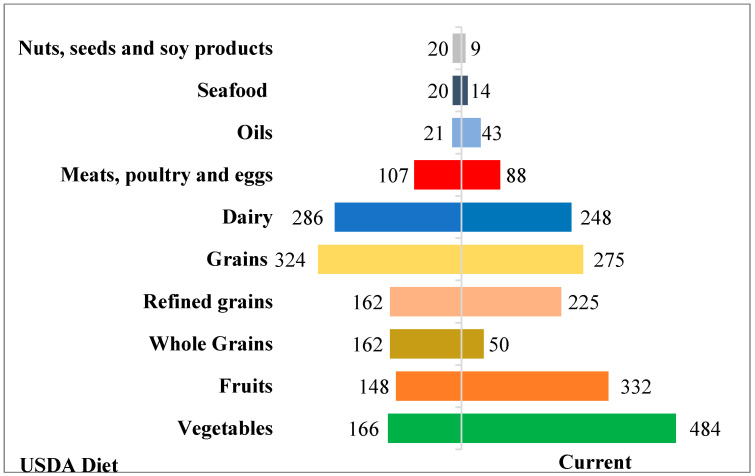
Comparison of current food groups intakes with USDA recommendations.

The comparison of the food groups’ consumption in the present population to the USDA Diet recommendations is represented in the figure below (Figure 2)

Vegetables, fruits, oils and refined grains consumption was higher in the present sample (484, 332, 43, and 225 g respectively) than USDA recommendations (166, 148, 21, and 162 g, respectively), while other food groups consumption was lower: whole grains (50 vs. 162 g); nuts, seeds and soy products (9 vs. 20 g); meats, poultry and eggs (88 vs. 107 g); dairy 248 vs. 286.

#### 3.2.3. Comparison of the Food Groups Consumed to the Mediterranean Diet Recommendations

**Figure 3:**
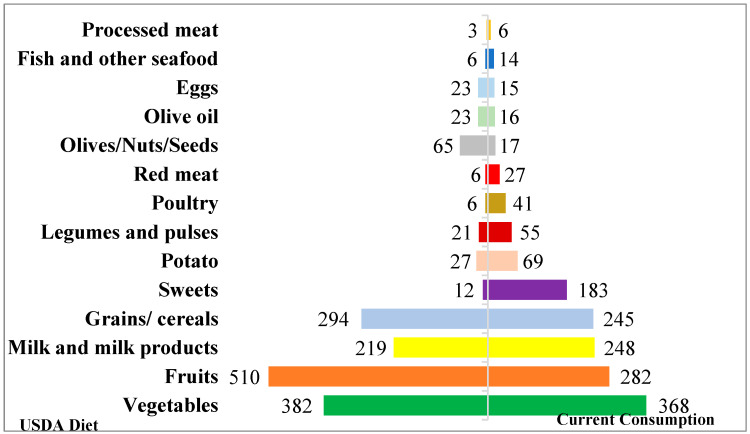
Comparison of current food groups intakes with Mediterranean Diet recommendations.

The comparison of the food groups consumed to the Mediterranean diet recommendations is illustrated in the graph below (Figure 3)

When compared to the Mediterranean diet recommendations, fruits (282 vs. 510 g), vegetables (168 vs. 382 g), olives/nuts/seeds (17 vs. 65 g), eggs (15 vs. 23), grains (245 vs. 294 g), and olive oil (16 vs. 23) consumption were lower. On the other hand, potato (69 vs. 27 g), legumes and pulses (55 vs. 21 g), sweets (183 vs. 12), poultry (41 vs. 6 g), red meat (27 vs. 6 g), processed meat (6 vs. 3 g), fish and seafood (14 vs. 6 g) and milk products (248 vs. 219 g) consumptions were higher.

### 3.3. Energy Content of Food Consumed

All participants did not meet the energy requirements for their trimester and age group. The highest adherence was around 84% observed during the first trimester of both age groups (Table 4).

### 3.4. Macronutrient Content of Food Consumed

No participant met the protein requirements in terms of the percentage of daily value. It was highest in the first trimester and decreased in the second and third. As for carbohydrates, women almost met the requirements (92%) while for fats and sugars, participants exceeded them. In detail, intakes of saturated fat were slightly above the requirements while those of unsaturated fats were not met. As for fibers, women did not meet all requirements and intake decreased from the first towards the end of the pregnancy. No significant difference was found between age and trimester categories (Table 5).

### 3.5. Micronutrient Content of Food Consumed

For most micronutrients, the recommended intakes were not met. Particularly, when comparing micronutrients intake to daily value, the highest intake of vitamin D was 11%, while that of iodine was 7.9%. Regarding iron and calcium, the highest intakes meeting the recommendations were 59.7% and 34.4% respectively, across all age groups and trimesters. As for vitamin A, 63.4% met the requirements from food among those aged 18–31 years, while only 54.2% met them among women aged 31–50 years. As for vitamin E, 52.6% and 51.9% met the recommended intakes among the younger and older age groups, respectively. Choline intake was reported for only 41.1% and 36.8% of women aged 18–30 years and 31–50 years, respectively. Zinc was consumed from food by 61.4% and 60.7% of younger and older aged groups, respectively. Around 18–24% of women aged 18–31 years and 20–35% of women aged 31–50 years did not consume the adequate amounts of folate from food. Over 20–32% of younger women and 29.2–37.3% of older women consumed inadequate amounts of cobalamin (vitamin B12). No significant difference was found between age and trimester categories (Table 6).

**Table 4:** Energy intake according to age and trimester categories.

			18–30 Years (N = 329)							31–50 Years (N = 171)					
	First Trimester (N = 76) Mean ± SD	%EER	Second Trimester (N = 164) Mean ± SD	%EER	Third Trimester (N = 89) Mean ± SD	%EER	*p*-Value ^1^	First Trimester (N = 24) Mean ± SD	%EER	Second Trimester (N = 95) Mean ± SD	%EER	Third Trimester (N = 52) Mean ± SD	%EER	*p*-Value ^1^	*p*-Value ^2^ (Between Age Groups)
EER ^a^	2000		2400		2600			1800		2200		2400			
Calorie intake Kcal	1688.0 ± 667.9	84%	1612.8 ± 530.0	67%	1645.9 ± 464.1	63%	0.609	1550.1 ± 500.2	86%	1660.0 ± 532.2	75%	1595.9 ± 494.0	66%	0.576	0.782

^a^ %EER = Energy Content/EER × 100. *p*-value < 0.05 is significant. Abbreviations: d day, EER Estimated Energy Requirement, kcal kilocalories, N number of participants, SD Standard Deviation. ^1^ Test: One-way ANOVA. ^2^ Test: Independent samples *t*-test.

**Table 5:** Macronutrient content of food consumed by Lebanese pregnant women and the percent contribution to daily value per age and trimester for women (N = 500).

			18–30 Years (N = 329)								31–50 Years (N = 171)						
	First Trimester (N = 76) Mean ± SD	%DV	Second Trimester (N = 164) Mean ± SD	%DV	Third Trimester (N = 89) Mean ± SD	%DV	Recommendations	*p*-Value ^1^	First Trimester (N = 24) Mean ± SD	%DV	Second Trimester (N = 95) Mean ± SD	%DV	Third Trimester (N = 52) Mean ± SD	%DV	Recommendations	*p*-Value ^1^	*p*-Value ^2^ (Between Age Groups)
Recommendations (Caloric intake source of goal kcal)	2000		2400		2600				1800		2200		2400				
Caloric intake kcal	1688 ± 667.9		1612.8 ± 530		1645.9 ± 464.1			0.609	1550.1 ± 500.2		1660 ± 532.2		1595.9 ± 494			0.576	0.782
Protein g	60.9 ± 23.1	85.8%	59.8 ± 17.3		56.2 ± 14.8		71	0.193	57.2 ± 13.7	80.5%	58.9 ± 17.1	82.9	57.5 ± 16.6	81	71	0.845	0.596
Protein (%)	14% ± 14%	64%	15% ± 13%	66%	14% ± 13%	61%	10 to 35		15% ± 11%	66%	14% ± 13%	63%	14% ± 13%	64%	10 to 35		
Carbohydrate g	213.3 ± 84.4	121.9%	198.3 ± 68.7		208.2 ± 66.8		175	0.279	187.3 ± 59.8	107	203.9 ± 72.8	116.5	209/3 ± 68.9	119.6	175	0.44	0.853
Carbohydrate (%)	51% ± 51%	92%	49% ± 52%	89%	51% ± 58%	92%	45 to 65		48% ± 48%	88%	49% ± 55%	89%	52% ± 56%	95%	45 to 65		
Fat (Total) g	65.7 ± 33.9		64.1 ± 25.7		65.5 ± 22.1			0.878	62.7 ± 25.1		66.9 ± 25.4		60.2 ± 22.5			0.274	0.798
Fat (%)	35% ± 46%	117%	36% ± 44%	119%	36% ± 43%	119%	25 to 35		36% ± 45%	121%	36% ± 43%	121%	34% ± 41%	113%	25 to 35		
Dietary Fiber g	20.5 ± 13.8	73.1%	18.4 ± 11.1	54	18.0 ± 6.9	50	14 g/1000 kcal	0.284	16.3 ± 6.3	65.3	18.9 ± 8.0	61.1	20.8 ± 12.3	61.1	14 g/1000 kcal	0.151	0.686
Fiber recommendation	28.0		34.0		36.0		14 g/1000 kcal		25		31		34		14 g/1000 kcal		
Sugar (Total) g	66.1 ± 35.8		67.4 ± 35.8		70.8 ± 34.5			0.665	63.4 ± 29.8		65.4 ± 31.4		68.3 ± 32.8			0.791	0.531
Sugar (%)	16% ± 21%	157%	17% ± 27%	167%	17% ± 30%	172%	<10%		16% ± 24%	164%	16% ± 24%	158%	17% ± 27%	171%			
Added Sugars g	6.4 ± 9.7		9.1 ± 13.2		11.1 ± 18.3			0.106	12.2 ± 25.6		8.9 ± 14		8.4 ± 14			0.605	0.887
Added sugars (%)	2% ± 6%	15%	2% ± 10%	22%	3% ± 16%	27%	<10%		3% ± 20%	32%	2% ± 11%	21%	2% ± 11%	21%	<10%		
Alcohol g	0.8 ± 6.9		1.5 ± 18.8		0.0 ± 0.0			0.709	0		1.8 ± 17.3		0.1 ± 0.4			0.685	0.948
Cholesterol mg	146.7 ± 99.1		143.8 ± 81.0		131.3 ± 83.9			0.442	134.2 ± 76.5		129.2 ± 90.4		123.5 ± 76.9			0.863	0.11
Saturated Fat g	19.0 ± 11.4		18.3 ± 8.4		18.4 ± 7.8			0.828	17.6 ± 6.4		18.3 ± 7.1		16.9 ± 7.0			0.55	0.372
Saturated Fat (%)	10% ± 15%	101%	10% ± 14%	102%	10% ± 15%	101%	<10%		10% ± 12%	102%	10% ± 12%	99%	10% ± 13%	96%	<10%		
Monounsaturated Fat g	22.3 ± 12.8		21.3 ± 10.3		23.3 ± 10.3			0.403	21.0 ± 9.7		21.4 ± 9.4		20.4 ± 9.0			0.805	0.318
Polyunsaturated Fat g	11.8 ± 7.9		11.7 ± 7.5		11.3 ± 5.5			0.887	11.9 ± 6.7		12.7 ± 8.7		11.3 ± 5.6			0.546	0.437
Linoleic PFA 18. 2 g	9.4 ± 6.6	72.30%	9.0 ± 6.8	89.7	8.8 ± 4.9	181.5	13	0.816	9.5 ± 5.7	72.9	10.1 ± 7.8	77.4	8.6 ± 4.8	65.9	13	0.441	0.426
Linolenic PFA 18. 3 g	0.8 ± 0.5	54.20%	0.7 ± 0.5	91.8	0.7 ± 0.3	202.6	1.4	0.934	0.7 ± 0.3	53.1	0.8 ± 0.9	58.2	0.8 ± 0.4	54.4	1.4	0.872	0.428
Trans Fatty Acid g	0.3 ± 0.4		0.3 ± 0.4		0.3 ± 0.4		0	0.903	04. ± 0.4		0.3 ± 0.2		0.3 ± 0.4		0	0.323	0.178

SD: standard deviation. Test: ^1^ Test: One-way ANOVA. ^2^ Test: Independent samples *t*-test *p* ≤ 0.05 was considered significant.

**Table 6:** Micronutrient content of food consumed by Lebanese pregnant women and the percent contribution to daily value per age and trimester for women (N = 500).

			18–30 Years (N = 329)								31–50 Years (N = 171)						
	First Trimester (N = 76) Mean ± SD	%DV	Second Trimester (N = 164) Mean ± SD	%DV	Third Trimester (N = 89) Mean ± SD	%DV	Recommendations	*p*-Value ^1^	First Trimester (N = 24) Mean ± SD	%DV	Second Trimester (N = 95) Mean ± SD	%DV	Third Trimester (N = 52) Mean ± SD	%DV	Recommendations	*p*-Value ^1^	*p*-Value ^2^ (Between Age Groups)
Sodium mg	21,533 ± 2149.3	93.6	1951 ± 788.3	85.1	1879 ± 804.6	81.7	2300	0.348	1828.3 ± 657.4	79.5	1912.4 ± 746.3	83.1	2017.4 ± 819.3	87.7	2300	0.557	0.639
Potassium mg	2452.7 ± 1011.6	84.6	2245.9 ± 878.2	77.4	2250.7 ± 675.6	77.6	2900	0.193	2212.9 ± 794.3	76.3	2268.4 ± 774.3	78.2	2328.8 ± 774.8	80.3	2900	0.817	0.839
Vitamin A RAE mcg	488.5 ± 282.5	63.4	426.5 ± 285.2	55.4	419.8 ± 214	54.5	770	0.182	376.7 ± 163.2	48.9	397.6 ± 195.4	51.6	417.5 ± 194.8	54.2	770	0.669	0.097
Vitamin C mg	84.5 ± 45.7	99.4	78.4 ± 44	92.1	82.1 ± 49.3	96.5	85	0.589	79.7 ± 43.1	93.8	80.3 ± 47	94.5	85.2 ± 50.6	100.3	85	0.816	0.821
Calcium mg	624.6 ± 243.2	62.5	627.3 ± 249.7	62.7	644.4 ± 293.5	64.4	1000	0.856	600.6 ± 164.3	60.1	619.7 ± 232.4	62	603.2 ± 239.7	60.3	1000	0.884	0.411
Iron mg	15.5 ± 6.3	57.5	15.2 ± 6.2	56.3	15.4 ± 5.8	57.1	27	0.915	14.5 ± 5.6	53.7	15.5 ± 6.9	57.4	16.1 ± 6.4	59.5	27	0.629	0.753
Vitamin D IU	65.5 ± 52.1	10.9	60.2 ± 46	10	65.7 ± 82	11	600	0.708	60.2 ± 42.1	10	55.4 ± 44.7	9.2	54.1 ± 44.5	9	600	0.85	0.143
Vitamin E mg	7.9 ± 6.5	52.6	7 ±4.6	46.4	7.5 ± 3.8	49.7	15	0.386	6.9 ± 4.5	45.8	7.8 ± 4.6	51.9	6.8 ± 3.2	45.3	15	0.336	0.715
Cobalamin (Vitamin B12) mcg	1.9 ± 1.1	72.3	2.1 ± 2	80	1.8 ± 0.9	68.2	2.6	0.301	1.9 ± 0.8	74.5	1.8 ± 1.1	70.8	1.6 ± 1.1	62.7	2.6	0.373	0.228
Vitamin K mcg	200.6 ± 132.1	222.9	164.3 ± 124.5	182.5	171 ± 142.7	190	90	0.133	156.4 ± 105.2	173.8	170.1 ± 126	189	189.3 ± 134.6	210.4	90	0.517	0.97
Phosphorus mg	898.3 ± 324.1	128.3	879.1 ± 278.6	125.6	858.4 ±276.1	122.6	700	0.675	830 ± 242.9	118.6	875.4 ± 257.6	125.1	277.4 ± 277.4	123.6	700	0.749	0.647
Iodine mcg	14 ± 27.8	6.4	17.3 ± 31.3	7.9	12.8 ± 22.8	5.8	220	0.437	17 ± 22.7	7.7	11.5 ± 23.6	5.2	14.2 ± 28.3	6.4	220	0.597	0.388
Magnesium mg	246 ± 94.9	70.3	230.9 ± 80.7	66	226.6 ± 69.4	64.7	350	0.272	221.4 ± 73.4	61.5	242.4 ± 84.2	67.3	245.4 ± 82.7	68.2	360	0.468	0.355
Zinc mg	6.7 ± 2.3	61.4	6.6 ± 2.3	60.4	6.4 ± 1.9	58.1	11	0.54	6.6 ± 1.9	60.4	6.7 ± 2.1	60.7	6.5 ± 2.3	59.4	11	0.926	0.892
Choline mg	185.1 ± 94.7	41.1	166.5 ± 64.6	37	160 ± 67	35.6	450	0.076	155.6 ± 55.7	34.6	157 ± 57.2	34.9	165.6 ± 70.4	36.8	450	0.679	0.143
Thiamin mg	1.5 ± 1.5	108.9	1.4 ± 1.1	96.6	1.3 ± 0.4	91.5	1.4	0.343	1.1 ± 0.4	81.9	1.2 ± 0.4	89.2	1.3 ± 0.4	94.8	1.4	0.193	0.191
Riboflavin mg	1.4 ± 0.8	96.4	1.3 ± 1.5	95.6	1.3 ± 1.5	96.4	1.4	0.997	1.1 ± 0.3	81.7	1.2 ± 0.4	84.6	1.2 ± 0.4	85.6	1.4	0.844	0.143
Niacin mg	16.3 ± 9.1	90.6	15.6 ± 5.7	86.6	14.8 ± 5.1	82.5	18	0.242	14.2 ± 4.7	78.8	15.1 ± 4.8	84	15.2 ± 4.5	84.3	18	0.655	0.335
Niacin Equivalent mg	24.2 ± 32.2	134.5	21.7 ± 21.2	120.7	18.5 ± 7	102.9	18	0.357	18.7 ± 6.8	103.6	18.8 ± 5.9	14.5	18.8 ± 6.2	104.5	18	0.994	0.121
Pyridoxine (Vitamin B6) mg	5.4 ±34.8	284.1	3.1 ± 24.1	163.6	1.2 ± 0.4	62	1.9	0.527	1.2 ± 0.4	65	1.3 ± 0.8	66.4	1.3 ± 1.0	69	1.9	0.911	0.313
Folate DFE mcg	495.3 ± 195/2	82.5	443.3 ± 161.3	73.9	459.8 ± 167.5	76.6	600	0.093	384.4 ± 118.6	64.1	434.1 ± 163	72.3	483.8 ± 172.7	80.6	600	0.413	0.405

SD: standard deviation. ^1^ Test: One-way ANOVA. ^2^ Test: Independent samples *t*-test. *p* ≤ 0.05 was considered significant.

## 4. Discussion

The present study is the most recent one with a representative sample size with an aim to evaluate the dietary intake of Lebanese pregnant women and compare it to recommendations. We evaluated food groups consumption as well as macro- and micronutrients. This is important especially amid rising food insecurity in Lebanon, which affects maternal and fetal health during the crucial period of pregnancy.

Previous findings reported that a healthy or prudent food consumption among pregnant women may lead to less complications and adverse child health outcomes [23]. Such a diet reduces the risks of pre-eclampsia, gestational hypertension, and gestational diabetes. A healthy diet can be summarized by being rich in vegetables, fruits, whole grains, nuts, legumes, fish, oils high in monounsaturated fat, and fiber. This diet should be low in fatty red meat and refined grains. Furthermore, healthy diets should avoid sugars, processed foods, and Trans and saturated fats [23].

Results showed that younger women had higher consumption of whole grains, refined grains, vegetables, fruits, nuts, seeds and soy products, oils, as well as meat, poultry and eggs. On the contrary, older women had higher intake of dairy and seafood. Furthermore, the present population did not meet all the recommended intakes for food group’s consumption according to USDA healthy pattern. In particular, vegetables, fruits, oils and refined grains consumption was higher in the present sample than USDA recommendations, while other food group’s consumption was lower: whole grains; nuts, seeds and soy products, meats, poultry and eggs; and dairy. Whole grains are good sources of fibers, vitamins and minerals, while refined grains lack the adequate amount of fibers and micronutrients and have been associated with higher odds of gestational diabetes [24]. Although protein intake is essential with some vitamins and minerals present adequately only in animal food, exceeding the amounts is detrimental, especially when consumed protein sources are high in fat and when the types of meat consumed are processed [25] On a positive side, vegetables intake that was higher than the recommendations, provides fibers and various vitamins, minerals and protective compounds [22]. Similarly to our results, a recent study among pregnant women in Arab countries showed that the majority did not adhere to USDA recommendations across all groups, expect for fruits [26]. Another cross-sectional study among 1939 Eastern Mediterranean postpartum women showed that the majority reported a low-no adherence to USDA recommendations (84%), in line with our study, across the five food groups [27]. It has been proven [22] across various populations and demographic groups that a diet rich in fruits, vegetables, whole grains, legumes, non- or low- fat dairy, lean meats and poultry, seafood, nuts, and unsaturated vegetable oils is associated with better health outcomes.

On the other hand, when compared to the Mediterranean diet recommendations, fruits, vegetables, olives/nuts/seeds, eggs, grains, olive oil consumption were lower, hence recommended intakes not met. On the other hand, potato, legumes and pulses, sweets, poultry, red meat, processed meat, fish and seafood and milk products consumptions were higher. Similarly to our results, a recent systematic review [28] of 50 studies in the Mediterranean region showed a low to moderate adherence to MedDiet in majority. According to the MedDiet pyramid, fruits, vegetables, nuts and seeds, olive oil and whole grains/cereals, which are plant-based foods, are located at the base of the diet and should be consumed daily. They provide essential nutrients and fibers, and therefore protect humans from diseases and improve their wellbeing [29]. In fact, adhering to the MedDiet is associated with lower risks of cardiovascular disease, heart attack, stroke, various types of cancer, Parkinson’s disease, Alzheimer’s disease, type 2 diabetes, rheumatoid arthritis and nonalcoholic fatty liver [30]. Another meta-analysis [31] of four randomized controlled trials among 2227 pregnant women found that adherence to MedDiet was associated with a lower risk of gestational diabetes and of a high gestational weight gain. Particularly, Seafood consumption is essential to provide favorable measures of cognitive development in young children. Weekly consumption (8 to 12 ounces) of low methylmercury sources is encouraged for women [22]. Other types of food in the MedDiet pyramid should be consumed in moderate amounts and at times should be left only for special occasions, namely red meat and sweets, which were consumed at excessive amounts in our sample. In fact, the Academy of Nutrition and Dietetics stated that pregnant women should reduce the consumption of high-added sugar in order to avoid excessive calories and maintain nutrient adequacy [32]. Moreover, adhering to the recommendations of limited added sugar intake was associated with lower offspring fat mass, lower maternal gestational weight gain, and lower risk of gestational diabetes mellitus [33,34] In a multicenter randomized trial among pregnant women with metabolic risk factors (ESTEEM), a Mediterranean-style diet had the potential to reduce gestational weight gain and the risk of gestational diabetes [35]. As for red meat, the consumption of processed red meat is associated with increased risks of diabetes, cardiovascular disease and colorectal cancer, while inconsistencies still cover unprocessed red meat [25]. In a recent study, higher consumption of red meat was associated with higher levels of anxiety and depression through associations with uric acid levels [36]. In general, a diet high in refined grains, fat, added sugars and low intake of fruits and vegetables was found to be associated with a higher risk of gestational diabetes [24].

As per the energy requirements for their trimester and age group, no one met the requirements. Regarding the evaluation of macronutrients intakes, protein, unsaturated fats and fibers requirements were not met, while those of fats and sugars were exceeded. As for micronutrients, recommended intakes were not fully met. Particularly, intakes of vitamin D and iodine were very low, in addition to relatively low adherence to recommendations for iron, calcium, vitamin A, E zinc and choline. A third did not meet the recommended intakes of folate and vitamin B12. The present study yielded macronutrients intake that were similar to another Lebanese study [11]. However, our energy intake was lower (Supplementary Table S1 for details). In the present sample, protein intake was insufficient among pregnant women. A recent study in the USA found that one in eight women in the second and third trimesters of pregnancy had inadequate intake of protein [37]. Protein intake is essential to promote adequate fetal growth [38]. A diet should be balanced with macronutrient intake to assure the best chance for a healthy pregnancy and optimal perinatal outcomes [23]. Moreover, any diet should avoid totally restricting any macronutrient. Women in the present sample exceeded the intakes of fats, similarly to another study among Spanish and Polish women [39]. Our sample reached the maximal recommended amounts of saturated fats, which should be limited to 10% of caloric intake [22]. The current study showed that unsaturated fats amount was not sufficient. Particularly, monounsaturated fats were also insufficiently consumed, although the MedDiet encourages the intake of olive oil, with is a major source of monounsaturated fats. On the contrary, the Spanish pregnant women evaluated in the study by Iglesias-Vazquez [39], consumed enough monounsaturated fats, probably due to the adoption of a Mediterranean diet, rich in olive oil consumption. Moreover, the excessive amounts of sugar consumed by our present sample should be limited to less than 10% of caloric intake [22]. Sugar consumption in our study (68–70.8 g maximal amount) was lower than that of the Jordanian study sample (125–137 g) [40], only slightly lower than the Spanish sample, but around twice higher than the Polish group [39] and higher than the ECLIPSE study sample (41.4 g) [41]. On the other hand, fiber intake in our study was lower than the Jordanian women [40], like the Polish group and higher than the Spanish sample [39] and higher than the ECLIPSE study sample [41].

In the present study, most of the micronutrients were not consumed according to the recommended amounts. Moreover, the difference was not significant between trimesters and age groups. Particularly, intakes of vitamin D and iodine were low as most of the women in our sample did not meet them. Vitamin D is essential for the skeletal development and bone mineral content of the fetus [42]. It can mostly be provided through exposure to sunlight, in addition to oily fish, eggs and fortified food products [43]. Nevertheless, Lebanese women showed deficient levels in studies, which calls to assess the need for supplementation [44]. Iodine is important for fetal neurocognitive development. Women should consume more dairy products, eggs, seafood and adopt iodized salt to meet recommendations during pregnancy [22]. Choline intake was low as 59–64% did not meet the requirements. Choline intake is essential to replenish maternal stores and support the child’s brain and spinal cord growth and development. It can be found in dairy, eggs, meats, and some seafood, as well as beans, peas, and lentils [22]. Regarding iron and calcium, 40–70% of women did not meet the recommendations. Iron is a key nutrient supporting fetal development and its needs increase during pregnancy. Women can be encouraged to consume lean meats, poultry, and some seafood for Heme iron sources, as it is found in animal source foods and is more readily absorbed by the body. They can also be encouraged to eat beans, peas, lentils, and dark-green vegetables, in addition to fortified cereals as non-heme iron sources, as it is found in plant source foods. These should be consumed with vitamin C to enhance absorption [22]. As for calcium intake, being essential for bones development, consumption of milk and dairy products should be advised [22]. As for vitamin A, 37% did not meet the requirements from food among those aged 18–31 years, while around half of the older age group did not meet them either. As for vitamin E, around the half of women didn’t meet the requirements from food. Zinc intake was insufficient for around 40% of the sample. Around 18–24% of women aged 18–31 years and 20–35% of women aged 31–50 years did not consume the adequate amounts of folate from food. Folate is essential to prevent neural tube defects and supplementation is common around pregnancy. Consumption of dark-green vegetables and beans, peas, and legumes such as lentils is encouraged to provide adequate folate amount but supplementation is often recommended [22]. Over 20–32% of younger women and 29.2–37.3% of older women consumed inadequate amounts of cobalamin (vitamin B12). The latter is provided through animal food sources. Intakes of fibers, cholesterol, iron, calcium, folic acid and sodium were lower than a previous study among Lebanese women [11] (Supplementary Table S1 for details). In a study in Jordan [40], similar patterns were observed. Highly inadequate intakes were reported for vitamin D, E, B6, iron, iodine, magnesium and zinc [40]. In that same study, vitamins A and B12 intakes were reported to be below the requirements for more than 47.4–60.4% and 46.7–66% of their sample. Around one third of women in the Jordanian women reported inadequate intakes of thiamin, riboflavin, niacin, vitamin C, calcium, copper, phosphorus and selenium. As for the folate intake, 28–45.8% had inadequate intakes. In another study among African American pregnant women, intakes of for vitamin D, folate, iron, calcium, and choline throughout pregnancy were assessed. Data showed that the majority did not achieve recommended intakes for these nutrients [45]. Furthermore, in a sample of the ECLIPSE study among pregnant women [41], data showed that intakes of vitamin D, iron and folate were much lower than the recommendations (below 35%). Finally, in a study evaluating intakes of pregnant women from Poland and Spain [39], intakes of iron, vitamin D, and vitamin B9 were particularly insufficient (below 40%).

Following the results of the present study, efforts should be done to monitor pregnant women’s nutritional status and advise them to adopt an adequate and diversified diet that abides by the recommendations of USDA diet and MedDiet. It is essential to implement public health policies and strategies that encourage Lebanese pregnant women to pursue healthier eating habits as these are essential to promote a good health and wellbeing. This necessitates a collective multidisciplinary effort. In general, consumption of whole grains instead of refined grains should be advised, in addition to adequate fish/seafood and unsaturated oils such as olive oil, healthy oils and nuts. Moreover, fruits and vegetables consumption should be supported, in addition to milk and dairy products. On the contrary, excessive amounts of added sugar and processed and fatty meats should be discouraged. A sufficient intake of protein from healthy sources should be provided from lean meats and legumes. Levels of vitamin D, iodine, iron, calcium, vitamin A, vitamin E, zinc and choline should be monitored, in addition to folate and vitamin B12. A diversified balanced and adequate diet should provide all necessary micronutrients.

Pregnant women’s intake in micronutrients should be provided by diet. However, when this is insufficient, supplementation under supervision should be prescribed [46,47]. In this sense, in an aim to provide guidelines to pregnant women in Lebanon, Naja et al.’s study [44] pointed out the importance of awareness about supplementation in essential micronutrients, given that needs increase during this period. These nutrients include Iron, and Folic Acid as required supplements, in addition to Vitamin D in case of lack of exposure and Omega-3 in case of lack of consumption of seafood.

## 5. Strengths and Limitations

The present study has the strength of showcasing an extended nutritional evaluation on a representative sample of the population. The chosen sample consists of pregnant women, which yields a high importance, due to the critical fetal and newborn outcomes. Analysis provided both food groups assessment and macro and micronutrients intake. Limitations of the present study include recall biases of self-reported data; this could lead to inaccuracies in portions sizes and therefore under or over reporting of food consumption.

## 6. Conclusions

This study provided a detailed nutritional assessment for a relatively sample of Lebanese pregnant women. There was insufficient adherence to USDA and MedDiet recommendations, with namely lack of whole grains and fish and unsaturated oils, and excessive amounts of sugar, refined grains, and red and processed meats. Macro and micronutrients intake were also assessed and showed insufficient intake of protein, unsaturated fats, in addition to vitamin D, iodine, followed by iron, calcium, vitamin A, vitamin E, zinc and choline. A third also did not meet the recommended intakes of folate and vitamin B12. Pregnant women should be encouraged to consume a balanced diet, rich in the components of a USDA healthy patterns and a MedDiet pattern, rich in whole grains instead of refined ones, lean meat instead of processed meat, fish, vegetables and fruits. They should also be encouraged to reduce the intake of added sugar. The present results highlight the urgent need for targeted nutritional interventions and public health initiatives to improve dietary practices among pregnant women in Lebanon.

## Figures and Tables

**Table 1:** Socio-demographic characteristics of the population.

		N (Total Number)	%
Age Category	18–30 years 31–50 years	329 171	66% 34%
Current Residency	Beirut/Mount Lebanon Northern Lebanon Southern Lebanon/Beqaa	246 100 154	49.2% 20% 3.8%
Crowding Index	1 or less >1	490 10	98% 2.00%
Mother Educational Level	Did not attend school Primary Middle Secondary University	4 14 54 102 326	0.8% 2.80% 10.80% 20.40% 65.20%
Spouse Educational Level	Did not attend school Primary Middle Secondary University	21 22 74 125 258	4.2% 4.40% 14.80% 25.00% 51.60%
Current Occupation	Not Working Full-time job Part-time job Self-employed	208 201 60 31	41.6% 40.20% 12.00% 6.20%
Monthly Household Income	None Less than 100$ 100–300$ >300$ <1,500,000 LBP 1,500,000–3,000,000 LBP >3,000,000 LBP	4 49 197 205 5 21 19	0.8% 9.80% 39.40% 41.00% 1.00% 4.20% 3.80%
Salary after crisis	Yes, my salary did not change No, half the salary No, less than half the salary No, I remain with no salary at all I already have no salary My salary increased	134 78 34 67 103 84	26.8% 5.60% 6.80% 13.40% 20.60% 16.80%
Number of children	0 1 2–3 >3	226 168 98 8	45.2% 33.60% 19.60% 1.60%
Pregnancy Trimester	First Trimester Second Trimester Third Trimester	100 259 141	20% 51.80% 28.200%

**Table 2:** Food groups consumption (grams/day) according to age groups (N = 500).

Food Groups Intake (g/day)	18–30 Years (N = 329) Mean ± SD	31–50 Years (N = 171) Mean ± SD	*p*-Value ^1^
Whole Grains	53.8 ± 161.38	43.77 ± 44.31	0.293
Refined Grains	231.38 ± 126.55	212.03 ± 109.11	0.09
Vegetables	494.66 ± 198.57	461.17 ± 192.78	0.071
Fruits	291.28 ± 200.92	278.69 ± 189.54	0.491
Nuts, Seeds and Soy products	9.23 ± 10.86	8.14 ± 8.87	0.256
Dairy	246.03 ± 175.58	250.85 ± 194.05	0.779
Oils	44.04 ± 24.87	40.95 ± 19.51	0.129
Meat, Poultry and Eggs	89.25 ± 58.3	82.72 ± 44.42	0.2
Seafood	12.79 ± 19.26	16.86 ± 35	0.16

SD: standard deviation. Test: ^1^ independent samples *t*-test. *p* ≤ 0.05 was considered significant.

**Table 3:** Food groups consumption (g/day) according to age groups and trimesters (N = 500).

		18–30 Years Mean ± SD				31–50 Years Mean ± SD			
	First Trimester	Second Trimester	Third Trimester	*p*-Value ^1^	First Trimester	Second Trimester	Third Trimester	*p*-Value ^1^	*p*-Value ^2^ (Per Age)
Whole Grains	38.78 ± 47.53	40.94 ± 45.6	90.34 ± 299.06	0.04 *	37.83 ± 38.74	46.24 ± 48.36	42.01 ± 39.06	0.67	0.293
Refined Grains	229.1 ± 115.90	230.7 ± 143.61	234.6 ± 100.14	0.96	207.8 ± 81.97	205.36 ± 105.01	226.16 ± 126.63	0.534	0.09
Vegetables	523.91 ± 189.18	490.41 ± 207.14	477.5 ± 189.54	0.3	441.28 ± 174.55	458.89 ± 203.32	474.52 ± 183.21	0.774	0.071
Fruits	309.03 ± 191.19	291.55 ± 198.92	275.62 ± 213.32	0.57	367.64 ± 319.47	249.2 ± 160.85	291.52 ± 143.27	0.019 *	0.491
Nuts, Seeds and Soy products	7.86 ± 9.31	10.05 ± 12.08	8.91 ± 9.62	0.33	7.83 ± 7.48	8.63 ± 9.93	7.38 ± 7.34	0.705	0.256
Dairy	235.74 ± 168.41	250.47 ± 186.33	246.64 ± 162.17	0.83	275.05 ± 177.97	245.4 ± 189.64	249.62 ± 211.25	0.8	0.779
Oils	45.11 ± 24.70	43.16 ± 25.59	44.74 ± 23.84	0.81	44.05 ± 22/04	37.85 ± 17.53	45.18 ± 21.06	0.065	0.129
Meat, Poultry and Eggs	85.62 ± 58.39	89.49 ± 54.63	91.89 ± 64.94	0.79	89.23 ± 55.33	83.48 ± 44.94	78.32 ± 37.87	0.593	0.2
Seafood	12.74 ± 14.66	13.28 ± 13.78	11.93 ± 29.09	0.87	13.75 ± 19.86	14.3 ± 16.11	22.96 ± 58.08	0.322	0.16

SD: standard deviation. ^1^ Test: One-way ANOVA. ^2^ Test: Independent samples *t*-test. * *p*-value < 0.05 is significant.

## Data Availability

The original contributions presented in the study are included in the article, further inquiries can be directed to the corresponding author.

## Supplementary Materials

The following supporting information can be downloaded at: https://www.mdpi.com/article/10.3390/nu16234059/s1, Table S1. Table comparing selected nutrients intake to a Lebanese study.
