# Supplementary material for: Energy, Macronutrients and Micronutrients Intake Among Pregnant Women in Lebanon: Findings from the Updated Lebanese National Food Consumption Survey (LEBANON-FCS)
Source: Nutrients. 2024 Nov 26;16(23):4059. doi: 10.3390/nu16234059 (PMC11643267; doi:10.3390/nu16234059)
Supplement: Supplementary file 1 [file nutrients-16-04059-s001.zip › nutrients-3303586-supplementary.pdf]

Table S1. Table comparing selected nutrients intake to a Lebanese study [11]

	**Current study**	**Other study** [11]
Energy kcal	1632	2381
CHO g	204	299.5
CHO kcal	50	51.1
Protein g	59	87.5
Proteins %	14	14.6
Fats g	65	98
Fats %	36	35.7
Sugars g	67	87
Fibers g	19	23.1
Cholesterol g	134 mg	198
Iron mg	15.4	18.3
Calcium mg	621.6	881
Vitamin D ug	1.58ug (59.31 IU)	10.6
Folic acid ug	83	352
Sodium mg	1957	5184
